# *phytanoyl-CoA dioxygenase domain-containing protein 1* plays an important role in egg shell formation of silkworm (*Bombyx mori*)

**DOI:** 10.1371/journal.pone.0261918

**Published:** 2021-12-30

**Authors:** Anli Chen, Pengfei Liao, Qiongyan Li, Qiaoling Zhao, Mengjie Gao, Pingyang Wang, Zenghu Liu, Gang Meng, Zhanpeng Dong, Min Liu

**Affiliations:** 1 The Sericultural and Apicultural Research Institute, Yunnan Academy of Agricultural Sciences, Mengzi, Yunnan, China; 2 The Key Sericultural Laboratory of Shaanxi, Ankang University, Ankang, Shaanxi, China; 3 The Sericultural Research Institute, Jiangsu University of Science and Technology, Zhenjiang, Jiangsu, China; Guizhou University, CHINA

## Abstract

Yun7^*Ge*^ is a giant egg mutant found in the silkworm variety Yun7. In comparison with the giant mutant *Ge*, the eggs of Yun7^*Ge*^ are larger. The number of laid eggs and hatching rate of Yun7^*Ge*^ are reduced, which is not conducive to reproduction. In this work, the target gene controlling giant egg trait is located on the Z chromosome and was determined through genetic analysis. Transcriptome results showed that *phytanoyl-CoA dioxygenase domain-containing protein 1* (*PHYHD1*) on the Z chromosome was silenced, and the 25 chorion genes on chromosome 2 were remarkably downregulated. Sequence analysis showed that the 73.5 kb sequence including the *PHYHD1* was replaced by a ~3.0 kb sequence. After knocking out the *PHYHD1* by using CRISPR/Cas9, the chorion genes were significantly downregulated. Hence, the silencing of *PHYHD1* leads to the downregulation of many chorion protein genes, thus directly causing giant eggs.

## 1. Introduction

The eggshell of *Bombyx mori* plays a significant role in protecting the developing embryo from external environmental hazards after oviposition. The eggshell is mainly comprised of chorion proteins, which are synthesized and secreted only by follicular cells located in a series of eight pupal ovarioles in *B*. *mori* [1]. Therefore, the egg shape is completely determined by the genotype of the female moth rather than that of the male moth, and this inheritance of egg traits is called “pseudo-maternal inheritance”. The morphology of *B*. *mori* eggs, such as kidney eggs (*ki*) [2], ellipsoid egg (*elp*) [3], new, small egg (*sm-n*) [4], and “Ming” lethal egg (*l-e*^*m*^) [5, 6]. All the chorion genes of *B*. *mori* are located on chromosome 2 [1], but not every mutant is caused by mutations of chorion genes. For instance, the gene responsible for the formation of *elp* eggs is located on chromosome 18 [3], and the *BmEP80* gene responsible for the change of eggshell structure of *l-e*^*m*^ mutant is located on chromosome 10 [6]. Hence, these non-chorion genes may indirectly affect the eggshell structure during egg formation.

**Fig 1:**
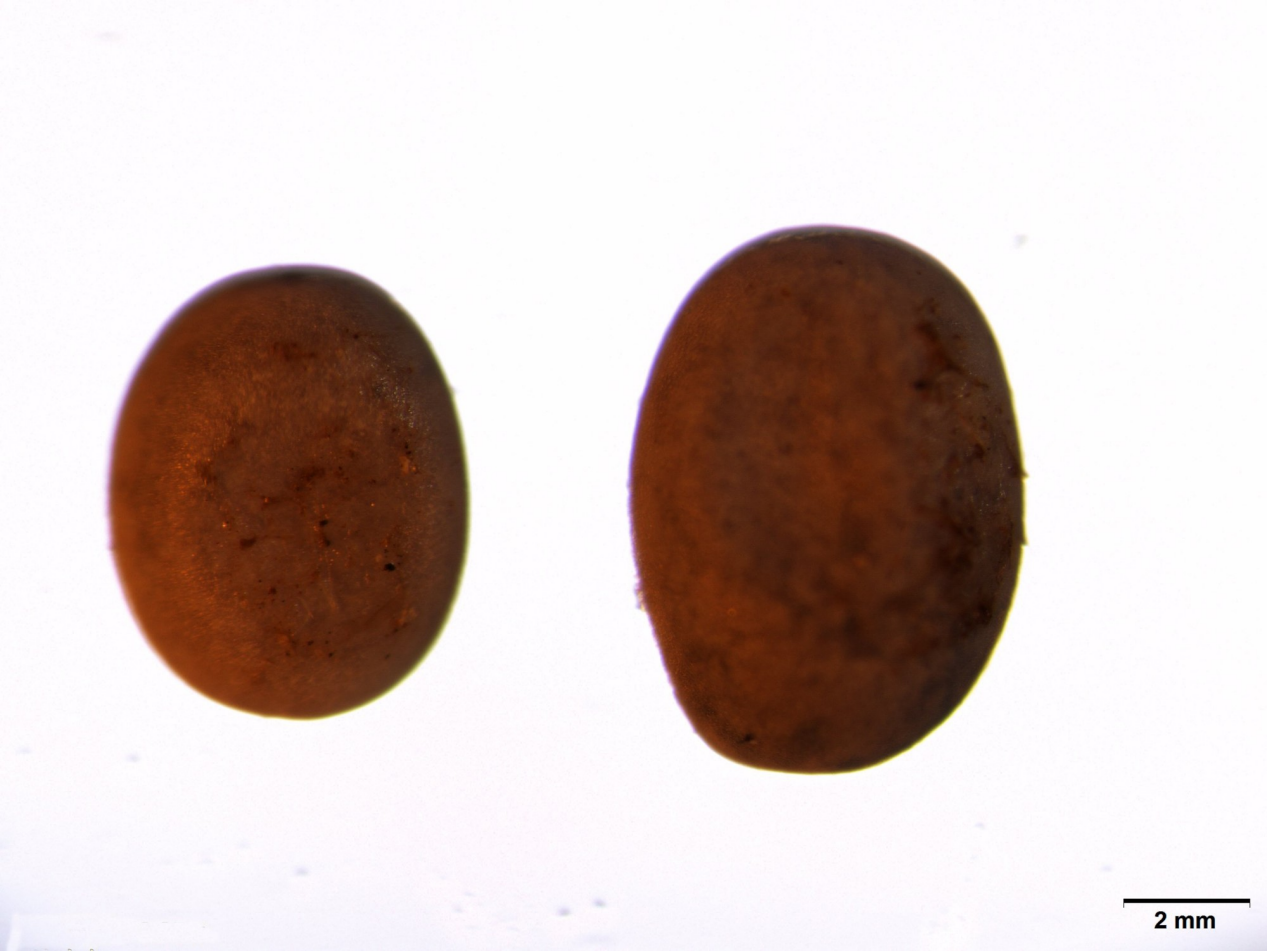
Egg size of Yun7 and Yun7^*Ge*^. Eggs of Yun7^*Ge*^ are approximately 71% heavier than the eggs of Yun7 (1000 eggs of Yun7 weigh 0.5242 g, and 1000 eggs of Yun7 ^*Ge*^ weigh 0.9008 g).

*Ge* is one kind of egg mutants, and the *Ge* locus is linked to the sex chromosome Z (chromosome 1) [7, 8]. Hence, the gene responsible for the giant eggs of *Ge* is not a chorion gene. Yun7^*Ge*^ was discovered in 2010 from the original seed of the silkworm variety Yun7 (Fig 1). In comparison with *Ge*, the eggs of Yun7^*Ge*^ are larger (eggs of Yun7^*Ge*^ are approximately 71% heavier than the eggs of Yun7, in which 1,000 eggs of yun7 weigh 0.5242 g, and 1,000 eggs of Yun7^*Ge*^ weigh 0.9008 g, and eggs of *Ge* are approximately 44% heavier than normal eggs [9]). The egg of Yun7^*Ge*^ is a short ellipse, grayish-purple, and abnormally larger than normal eggs. Female moths of Yun7^*Ge*^ lay fewer eggs than normal female moths, and eggs of Yun7^*Ge*^ have a low hatching rate (~40%). The newly-hatched larvae of Yun7^*Ge*^ are larger than the normal ones, but with the increase of instar, the size difference gradually reduced. By the fifth instar, the larvae size of Yun7^*Ge*^ is similar to that of the normal ones, and the cocoon size and weight are similar. In the present work, the chromosome where the target gene of Yun7^*Ge*^ is located was determined through genetic analysis, the target gene was determined through transcriptome sequencing and gene sequence analysis, and the function of the target gene was verified by CRISPR/Cas9.

## 2. Materials and methods

### 2.1 Silkworm strains and genetic analysis

Silkworm strains P50, Yun7, and its mutant Yun7^*Ge*^ were preserved by the Sericultural and Apicultural Research Institute, Yunnan Academy of Agricultural Sciences. All *B*. *mori* larvae were reared on fresh mulberry leaves at 25 ± 0.5°C and constant humidity of 75%–80%. The homozygote of Yun7^*Ge*^ was obtained after several generations of inbreeding, and then the reciprocal cross of Yun7^*Ge*^ and wild-type P50 were performed. The segregation of normal and giant eggs in F_1_ and its inbred progenies F_2_ and F_3_ was investigated.

Silkworm strain Qiufeng was preserved by the Sericultural Research Institute of Jiangsu University of Science and Technology. The eggs produced by Qiufeng were diapause eggs and needed to be treated with hydrochloric acid to terminate diapause. The microinjection destroys the integrity of the eggshell, and hydrochloric acid treatment causes the death of the embryo. Therefore, a low temperature of 17°C (normal incubation temperature, 25–26°C) was used for incubation to make the eggs laid by the offspring as diapause-free eggs [10].

### 2.2 RNA preparation and Illumina RNA-seq

Total RNA was prepared using TRIzol (Invitrogen, USA) from the mixture of three whole oviducts of Yun7 and Yun7^*Ge*^ before mating, and two replicates were set for each sample. RNA degradation and contamination were monitored on 1% agarose gel. RNA purity was checked using the NanoPhotometer^®^ spectrophotometer (IMPLEN, CA, USA). RNA concentration was measured using Qubit^®^ RNA assay kit in Qubit^®^2.0 Fluorometer (Life Technologies, CA, USA). RNA integrity was assessed using the RNA Nano 6000 assay kit of the Agilent Bioanalyzer 2100 system (Agilent Technologies, CA, USA).

A total amount of 1.5 μg RNA per sample was used as input material for the RNA sample preparations. Sequencing libraries were generated using NEBNext® Ultra™ RNA library prep kit for Illumina® (NEB, USA) following manufacturer’s recommendations, and index codes were added to attribute sequences to each sample. Briefly, mRNA was purified from total RNA by using poly-T oligo-attached magnetic beads. Fragmentation was carried out using divalent cations under elevated temperature in NEBNext first strand synthesis reaction buffer (5×). First strand cDNA was synthesized using random hexamer primer and M-MuLV reverse transcriptase (RNase H-). Second strand cDNA synthesis was subsequently performed using DNA polymerase I and RNase H. The remaining overhangs were converted into blunt ends via exonuclease/polymerase activities. After adenylation of the 3′ ends of DNA fragments, NEBNext Adaptor with hairpin loop structure was ligated in preparation for hybridization. To select cDNA fragments of preferentially 150–200 bp in length, we fragmented the library fragments with AMPure XP system (Beckman Coulter, Beverly, USA). Then, 3 μl of USER Enzyme (NEB, USA) was used with size-selected, adaptor-ligated cDNA at 37°C for 15 min, and then for 5 min at 95°C before PCR. Then, PCR was performed with Phusion high-fidelity DNA polymerase, universal PCR primers, and index (X) primer. At last, PCR products were purified (AMPure XP system), and library quality was assessed on the Agilent Bioanalyzer 2100 system.

The index-coded samples were clustered on a cBot cluster generation system by using TruSeq PE cluster kit v3-cBot-HS (Illumina) according to the manufacturer’s instructions. After cluster generation, the library preparations were sequenced on an Illumina Hiseq platform, and paired-end reads were generated.

The raw data (raw reads) of fastq format were first processed using in-house perl scripts. In this step, clean data (clean reads) were obtained by removing reads containing adapter, reads containing ploy-N, and low-quality reads from raw data. At the same time, the Q20, Q30, GC-content, and sequence duplication level of the clean data were calculated. All downstream analyses were conducted based on clean data with high quality. The raw data presented in this publication have been deposited in GEO (GEO accession: GSE173672).

### 2.3 RNA-seq data analysis

The silkworm genome sequence was obtained from SilkDB 3.0 (https://silkdb.bioinfotoolkits.net/) and KAIKObase (https://kaikobase.dna.affrc.go.jp/), and clean reads were aligned with reference genome sequences by using Tophat software [11]. Gene expression levels were calculated using the reads per kb per million reads (RPKM) method [12]. P value<0.05, false discovery rate (FDR) ≤ 0.001, and RPKM>5 were set as thresholds for gene expression. The expression levels of genes between Yun7 and Yun7^*Ge*^ were compared to screen differentially expressed genes (DEGs). Differential expression analysis of Yun7 and Yun7^*Ge*^ was performed using DEGseq (2010) [13] R package. P value was adjusted using q value [14]. Q value<0.005 and ∣*log* 2 (foldchange)∣>1 were set as the threshold for significantly differential expression. The DEG sequences were BLAST-searched, mapped, annotated, and analyzed using Kyoto Encyclopedia of Genes and Genomes metabolism pathways and Blast2GO software (version 2.7.2) according to the Blast2GO Tutorial [15].

### 2.4 Screening candidate genes

Candidate genes were screened according to the genetic analysis and DEGs of transcriptome analysis. Primers of candidate genes were designed according to the Silkworm genome database KAIKObase (https://kaikobase.dna.affrc.go.jp/). The genomic DNA of Yun7 and Yun7^*Ge*^ was extracted as previously described [16].The concentration and purity of genomic DNA were determined using an ultra-micro spectrophotometer (NanoPhotometer N60) at a 260/280 absorbance ratio, and the purified DNA was stored at −20°C.

The sequences of candidate genes were amplified using Yun7 and Yun7^*Ge*^ genomic DNA as templates, and the PCR products were cloned into pMD19-T vector (TaKaRa, Japan). The recombinant plasmids were transformed into *Escherichia coli* TOP10 competent cells. Sequencing was performed by Sangon Biotech (Shanghai) Co., Ltd, and the mutant genes were identified according to the sequence differences. The primer sequences are listed in S1 Table.

### 2.5 Chromosome walking

Specific primers at both ends of the mutation region of Yun7^*Ge*^ were designed for chromosome walking. The chromosome walking reaction was performed according to the instructions of the TaKaRa Genome Walking Kit (TaKaRa, Japan). 5 μl PCR products were used for 1% agarose gel electrophoresis, and the amplified products were cut and recovered using Takara MiniBEST Agarose Gel DNA Extraction Kit Ver. 4.0 (TaKaRa, Japan). Solution I in the DNA Ligation Kit (TaKaRa, Japan) was used to connect the recovered product to pMD 19 T Vector, and the recombinant plasmid is thermally transformed into competent cell TOP10. Sequencing was performed by Sangon Biotech (Shanghai) Co., Ltd. The primer sequences are listed in S1 Table.

### 2.6 sgRNA synthesis and microinjection

The sgRNA sites of the mutant genes were predicted using the online analysis software CRISPRdirect (http://crispr.dbcls.jp/), and the specific primer sequences (sequence: TTCTAATACGACTCACTATAG (N_20_) GTTTTAGAGCTAGA, N_20_ represented the target site) containing the T7 promoter (sequence with underline) were synthesized by Sangon Biotech (Shanghai) Co., Ltd (S1 Table). The sgRNAs was synthesized based on the protocol of EnGen^®^ sgRNA synthesis kit (New England Biolabs, USA). After purification and determination of concentration, sgRNAs and Cas9 protein (EnGen^®^ Spy Cas9 NLS, New England Biolabs, USA) were mixed and used for the microinjection of silkworm eggs.

A pair of sgRNA (1000 ng/μL each) and cas9 protein (20 μmol/L) were mixed by the ratio of 1:1 [17]. The mixture was injected into the eggs of Qiufeng (diapause-free eggs) within 2 h after oviposition through TransferMan 4r microinjection system (Eppendorf, Germany). Each silkworm egg was injected with approximately 5 nL of cas9-sgRNAs mixture. After injection, the silkworm eggs were incubated at 25°C and 75% relative humidity. After hatching, the larvae were reared in a conventional manner with fresh mulberry leaves. Female moths were mated with uninjected male moths, and the offspring of mating was recorded as F_1_. After oviposition, the genomic DNA of female moth was extracted according to the phenotype of the eggs. The specific primers designed at both ends of the sgRNA site were used for SNP typing on the CFX96^TM^ real-time system (Bio-Rad, USA), and the PCR products were sequenced of the abnormal high-resolution melt). The PCR reaction system was used according to the instructions of Precision Melt Supermix (Bio-Rad, USA).

### 2.7 Quantitative reverse transcriptase PCR

The male moths of the F_1_ generation were mated with the uninjected female moths, and the offspring was recorded as F_2_. The female moths of the F_2_ were mated with uninjected male moths, and the offspring was recorded as F_3_. The oviducts of F_3_ were dissected, and the total RNA of the oviducts was extracted according to the phenotype of the eggs.

The total RNA of three oviducts obtained from different female moths of Yun7 and Yun7^*Ge*^ was isolated using TRIzol (Invitrogen, USA). RNA was reverse transcribed using PrimeScript^TM^ RT reagent kit with gDNA eraser (TaKaRa, Japan) following the manufacturer’s instructions. The cDNA products were diluted by five-fold with ddH_2_O. Each 20 μL of the quantitative reverse transcriptase PCR (qRT-PCR) reaction system consisted of 10 μL of SYBR premix ex tag (FastStart Universal SYBR Green Master (ROX), Roche, Switzerland), 0.5 μL of the specific primers, 1 μL of cDNA template, and 8 μL of ddH_2_O, and three replicates were produced. qRT-PCR was performed in a StepOnePlus^TM^ real-time PCR system (Applied Biosystems, USA) with a two-step reaction protocol of 40 cycles of 94°C for 5 s and 60°C for 1 min. The housekeeping *B*. *mori* actin 3 gene (GenBank ID: NM_001126254) [18] was used as a reference to eliminate bias among samples, and qRT-PCR results were converted and calculated using the 2^-ΔΔ*Ct*^ method [19]. The primers used for qRT-PCR are shown in S1 Table.

## 3. Results

### 3.1 Genetic characteristics of Yun7^*Ge*^

The female genotype of silkworm is ZW, and the male genotype is ZZ. The gene that controls the giant egg trait of *Ge* is located on the Z chromosome (chromosome 1) [7]. To determine whether the gene controlling the giant egg trait of Yun7^*Ge*^ is located on the Z chromosome, we performed genetic analysis of Yun7^*Ge*^.

The F_1_ generation of the P50 female moth and Yun7^*Ge*^ male moth only laid normal eggs. The F_1_ generation self-bred to produce the F_2_ generation, and all the eggs laid by the F_2_ generation were giant eggs. The F_2_ generation self-bred to produce the F_3_ generation, and the ratio of the number of female moths that lay giant eggs to the number of female moths that lay normal eggs was 1:1 (Table 1). The F_1_ generation of the Yun7^*Ge*^ female moth and P50 male moth only laid giant eggs. The F_1_ generation self-bred to produce the F_2_ generation, and all the eggs laid by the F_2_ generation were normal eggs. The F_2_ generation self-bred to produce the F_3_ generation, and the ratio of the number of female moths that lay giant eggs to the number of female moths that lay normal eggs was 1:1 (Table 1).

**Table 1:** Separation of giant eggs and normal eggs in the progeny of P50 and Yun7^*Ge*^ reciprocal crosses.

Cross combinations	Generations	Batch number of giant eggs	Batch number of normal eggs	Segregation ratio	$\chi_{c}^{2}$
P50×Yun7^*Ge*^	F1	0	268	*n*	—
	F2	273	0	*n*	—
	F3	129	146	1:1	0.93
Yun7^*Ge*^×P50	F1	262	0	*n*	—
	F2	0	275	*n*	—
	F3	141	123	1:1	1.09

The segregation ratio in Table 1 appear only when the gene controlling giant egg trait is located on the sex chromosome Z. Hence, the gene controlling the giant egg trait is located on the sex chromosome Z. This result is consistent with that of *Ge* [7].

### 3.2 DEGs

Based on genetic analysis, the oviduct transcriptome of Yun7 and Yun7^*Ge*^ before oviposition was analyzed to determine the DEGs on Z chromosome. Values of ∣log 2∣>1 and FPKM>100 and whether the genes were up- or downregulated in giant eggs of the two replicates were used as the standard for screening DEGs. A total of 65 DEGs were screened. Among these DEGs, seven genes were upregulated, and 58 genes were downregulated in giant eggs. Genetic analysis showed that the gene controlling giant egg traits is located on Z chromosome. Hence, these DEGs were classified according to the chromosome where the genes are located. Results showed that chromosome Z has only one DEG, and it was almost not expressed in giant eggs (S2 Table). Interestingly, chromosome 2 had 35 DEGs, including 25 chorion genes, which were all downregulated (S2 Table). Considering the expression of chorion genes changed considerably, chorion genes with the value of FPKM<100 were analyzed. Results showed that three genes were upregulated, whereas 22 genes were downregulated in giant egg (S3 Table). Therefore, the gene mutation on the Z chromosome caused an important effect on the expression of the chorion genes on chromosome 2.

### 3.3 Determine the mutation site

**Fig 2:**
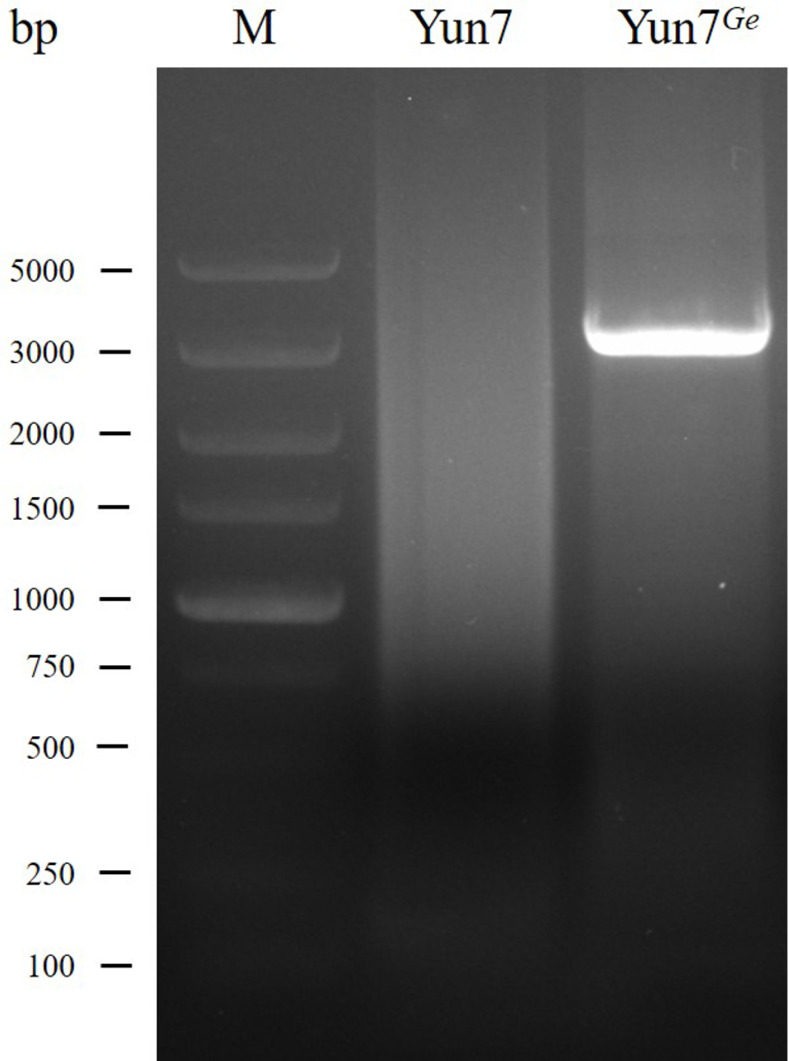
PCR results at both ends of the mutation region. The first lane indicates the DNA Marker DL5000, the second lane indicates the PCR product with the Yun7 genome as the template, and the third and fourth lanes indicate the PCR product with the Yun7^*Ge*^ genome as the template. When Yun7 genome was used as template, the target fragment was very large (~73.5 kb) to be amplified. When Yun7^*Ge*^ genome was used as template, the 73.5kb fragment was replaced by a 3kb fragment because of mutation, so it could be amplified.

Based on the analysis of the DEG on Z chromosome, the DEG was *phytanoyl-CoA dioxygenase domain-containing protein 1* (*PHYHD1*), which was consistent with the results of previous studies [20]. The gene number in KAIKObase (https://kaikobase.dna.affrc.go.jp/) is KWMTBOMO00542. To verify whether the *PHYHD1* gene is mutated, we designed multiple pairs of primers for PCR by using Yun 7 and Yun7^*Ge*^ genome as templates. One pair of primers (S1 Table) could not amplify with Yun 7 genomic DNA as template but can amplify a ~3 KB fragment with Yun7^*Ge*^ genomic DNA as template (Fig 2).

**Fig 3:**
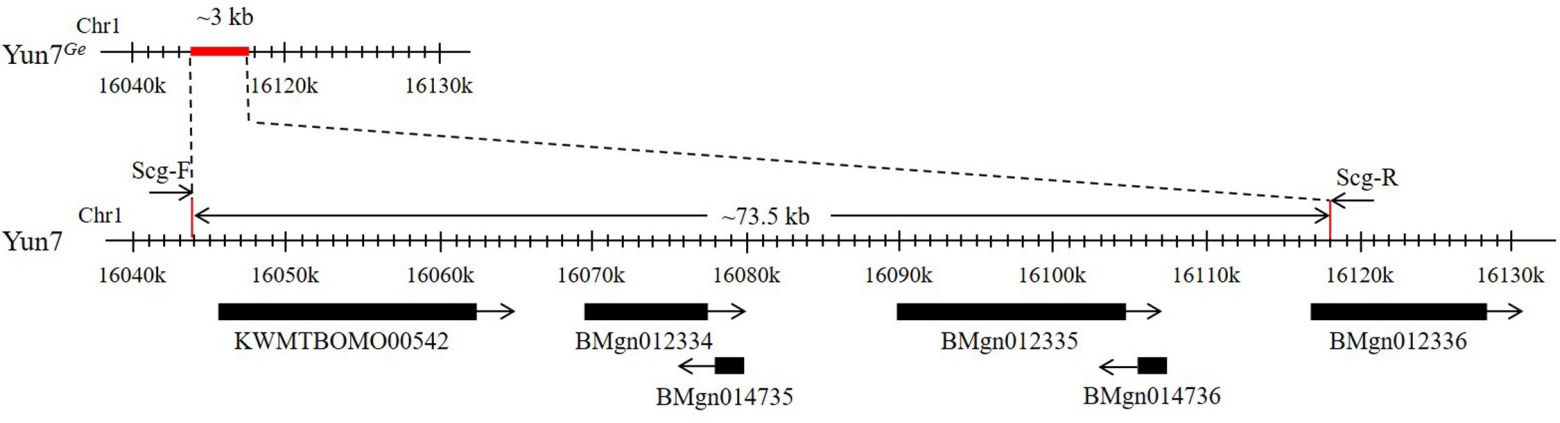
Mutation position of Yun7^*Ge*^. The 73.5 kb sequence of the six genes including *PHYHD1* was deleted and replaced by a ~3.0 kb sequence. Except for the partial deletion of BMgn012336, the five remaining genes were completely deleted.

The results showed that the 73.5 kb sequence of the six genes including *PHYHD1* was deleted and replaced by a ~3.0 kb sequence (Fig 3). The inserted fragment (~3.0 kb) was cloned into the pMD19-T vector for sequencing, and the mutation sites at both ends of the mutation region were determined (S1 Fig). However, considering the special structure of the inserted sequence, it could not be completely sequenced.

To determine the sequence information of the inserted fragment, chromosome walking was performed at both ends of the mutation region. The results showed that a ~2.3 kb sequence was obtained on one side of the mutation region (S2 Fig), but the target band was not obtained on the other side of the mutation region. The forward primer ScgF1 was designed with the ~2.3 kb sequence as a reference, and ScgR was used as the reverse primer for PCR amplification, and a product with a size of ~1300 bp was obtained (S3 Fig). After cloning and sequencing, the ~1300 bp PCR products were spliced with the ~2.3kb sequence. Finally, the sequence in the middle of the mutation sites at both ends was determined to be 3105 bp (S1 Sequence). Sequence alignment showed that the sequence was one of the missing 73.5 kb sequences, and no transposon or similar functional sequence was found. There is a high GC region in the sequence, which may be the reason for the failure of normal sequencing.

Based on the analysis of the transcriptome results of five genes other than *PHYHD1*, BMgn012334, BMgn014735, and BMgn012335 were slightly expressed (RPKM<10) in normal eggs but not expressed at all in giant eggs, and BMgn014736 and BMgn012336 were not expressed both in normal and giant eggs. Therefore, these five genes were excluded when screening for DEGs. These results indicate that the occurrence of giant eggs was most likely caused by the mutation of *PHYHD1*.

### 3.4 *PHYHD1* affects the expression of chorion protein genes

Giant egg is an ovoid mutation, and the formation of giant egg is closely related to chorion protein. Genetic analysis and transcriptome results showed that the mutation of *PHYHD1* located on chromosome 1 caused the silencing of the *PHYHD1*, but no evidence showed that the silencing of *PHYHD1* directly caused giant eggs. By contrast, the expression change of 50 chorion genes (47 genes were downregulated and 3 genes were upregulated, including the genes with FPKM>100 and FPKM<100) on chromosome 2 was more likely to cause the occurrence of giant eggs.

**Fig 4:**
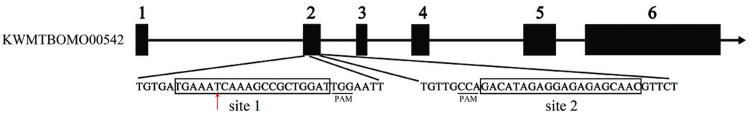
Knockout result of *PHYHD1* using CRISPR/Cas9. SgRNAs sites are shown in the box. The base pointed to by the red arrow is missing.

**Fig 5:**
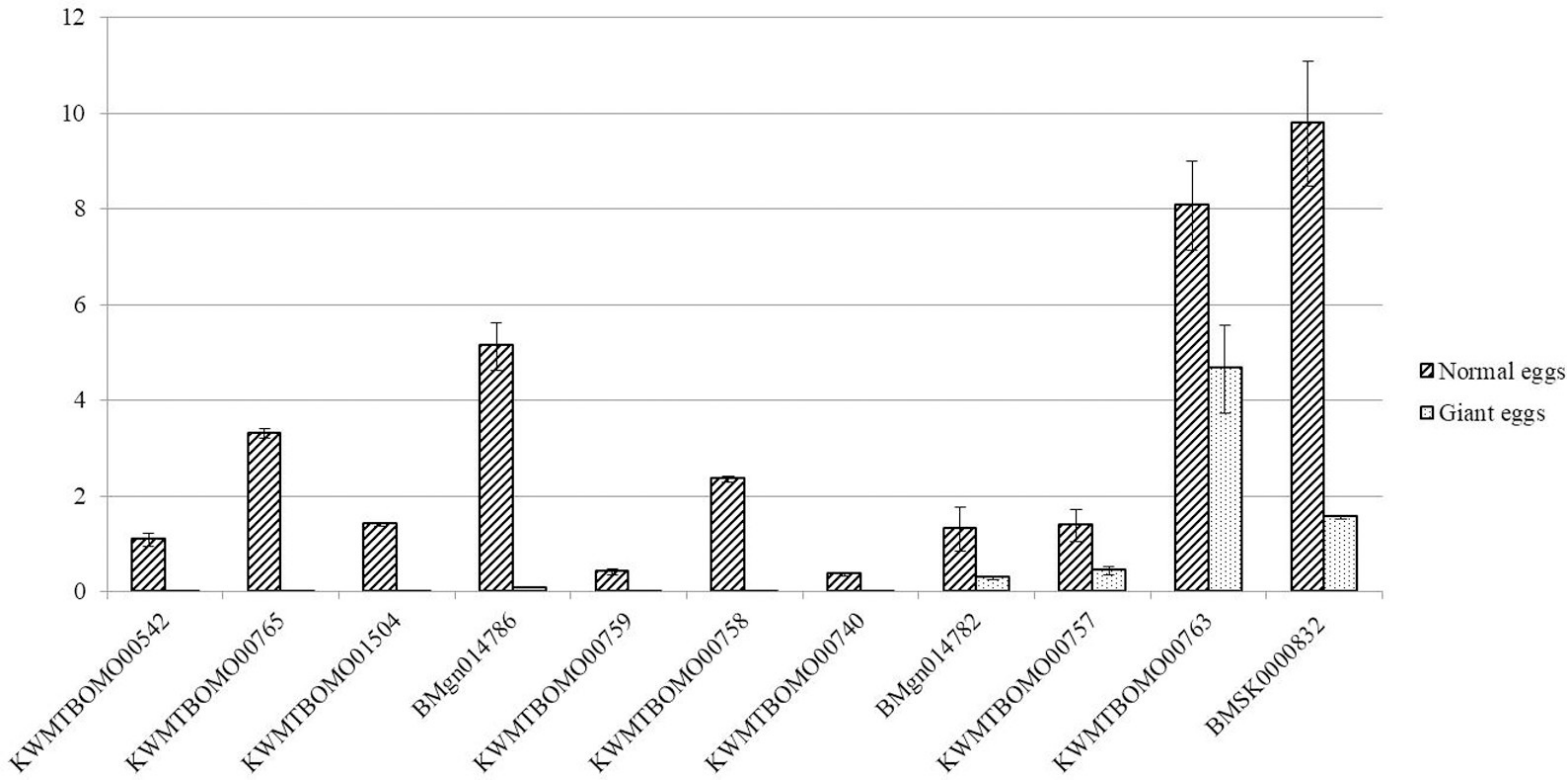
Phenotype of F_3_ generation after gene knockout. Compared with normal eggs, the egg shape of giant eggs of F3 is larger and the number of eggs is reduced.

To determine whether the downregulation of chorion genes is related to silencing of *PHYHD1*, we knocked out *PHYHD1* by CRISPR/Cas9. Two sgRNA sites in the exon2 of *PHYHD1* were predicted by the online analysis software CRISPRdirect (S1 Table). The female moths were mated with the uninjected male moths, and the results showed that the eggs (F_1_ generation) laid by one female moth were larger than normal eggs. SNP (S4 Fig) and sequencing analysis found that a base was missing in the second exon of *PHYHD1* resulted in a frameshift mutation (Fig 4). The male moths of the F_1_ generation were mated with the uninjected female moths, and the eggs laid by all female moths (F_2_ generation) were normal. The female moths of the F_2_ generation were mated with uninjected male moths, half of the female moths (F_3_ generation) laid normal eggs, and the other half of the female moths laid giant eggs (Fig 5).

**Fig 6:**
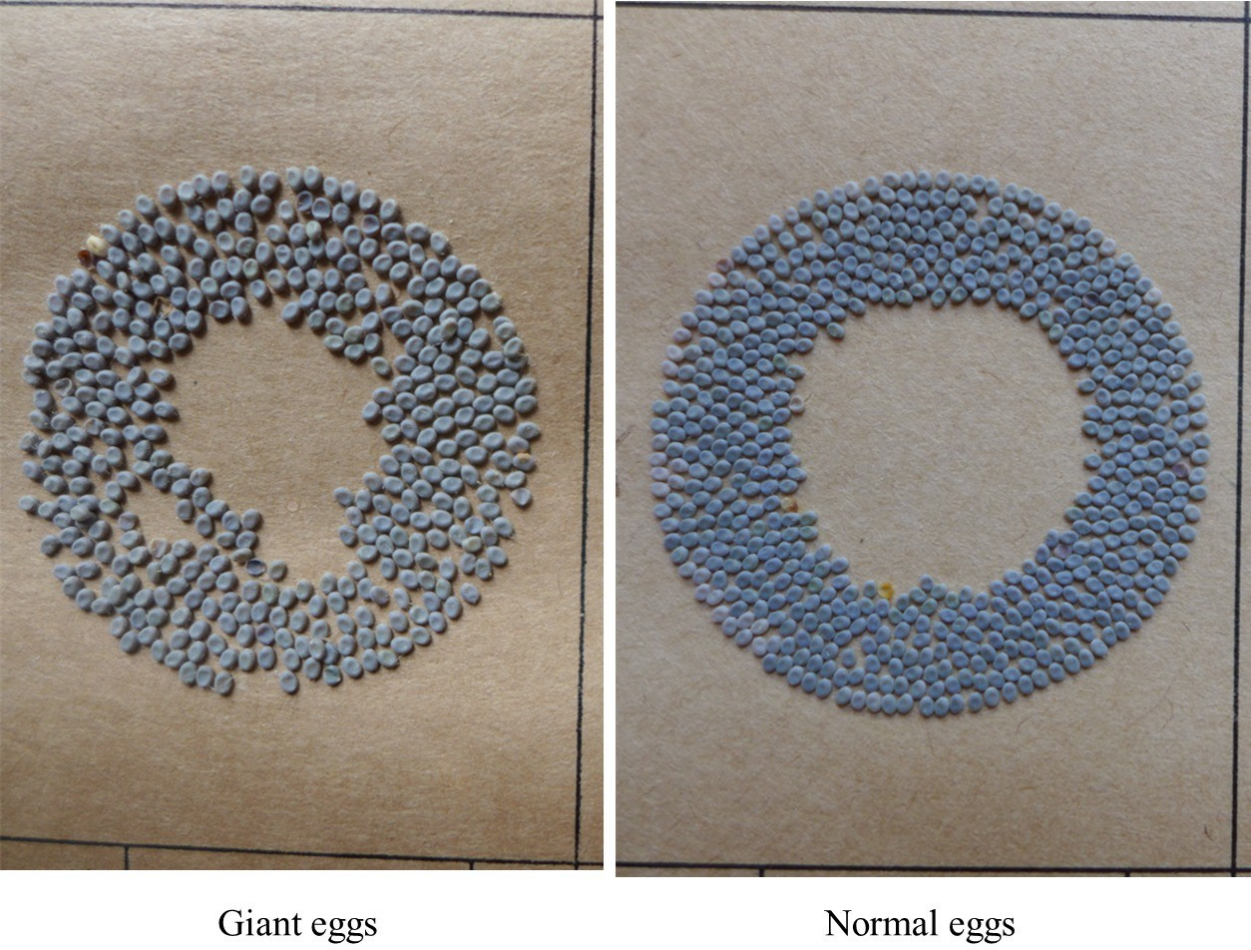
Expression of *PHYHD1* and 10 randomly selected chorion genes in normal and giant eggs (after *PHYHD1* knockout). *PHYHD1* (KWMTBOMO00542) was expressed normally in normal eggs but almost not expressed in giant eggs. All 10 chorion genes were significantly downregulated in giant eggs.

The expression results of *PHYHD1* and 10 randomly selected chorion genes in whole oviduct before mating showed that *PHYHD1* was expressed normally in normal oviduct but slightly expressed in oviduct that produce giant eggs, while the selected chorion genes were significantly downregulated in oviduct that produce giant eggs (Fig 6). The knockout of *PHYHD1* could downregulate the chorion protein genes, and the downregulation of chorion protein genes may directly direct cause giant eggs.

## 4. Discussion

The eggshell of *B*. *mori* mainly comprised of chorion proteins. So far, 127 chorion genes have been found, and all these genes are located in a region of chromosome 2 [1]. In the present study, the expression of 50 chorion genes changed after *PHYHD1* gene silencing, among which 47 genes were downregulated and three genes were upregulated. Knockout of *PHYHD1* showed that *PHYHD1* silencing could affect the expression of chorion genes.

Chorion proteins of *B*. *mori* are synthesized and secreted only by follicular cells located in a series of eight pupal ovarioles [21]. Developing follicles can be grouped along the length of the ovariole, from anterior to posterior, into three broad developmental periods, namely, previtellogenesis, vitellogenesis, and choriogenesis [22]. The follicle itself consists of a single layer of polyploid epithelial cells, namely, the follicular epithelium, which surrounds the oocyte [23]. During vitellogenesis, the oocyte accumulates yolk proteins, and its growth becomes accelerated with respect to the nurse cells [22]. The follicular epithelium becomes involved in the transport of vitellogenin, which is produced and secreted by the fat body from the hemolymph towards the oocyte; in addition, it remarkably contributes to the accumulation of yolk into the oocyte by producing and secreting the egg-specific protein (ESP) that will comprise up to one third of the total yolk protein in the oocyte [24, 25]. The transcriptome results of the present study showed that the *ESP* (KWMTBOMO11748) in giant eggs was significantly downregulated compared with normal eggs (S2 Table). *PHYHD1* might play a role in the hydroxylation of fatty acids and interact with some fatty acids or derivatives thereof [26]. The reference transcriptome data in silkworm *B*. *mori* also showed that *PHYHD1* was specifically and highly expressed in the fat body and ovary [27] (https://kaikobase.dna.affrc.go.jp/KAIKObase/bm_new_gene_desc/pages/KWMTBOMO00542.html). Hence, the mutation of *PHYHD1* may cause abnormal production and secretion of vitellogenin, resulting in abnormal production and secretion of yolk proteins.

The total weight of eggs laid by a normal female moth and a *Ge* female moth has no difference, and the yolk proteins and other components of giant eggs increase proportionally with the increase of the egg size [9]. The increase in yolk proteins increases the size of oocyte. As oocyte increases in size, more follicular cells are required to secrete chorion proteins to form eggshell. During the eggshell formation of *Ge*, the number of follicular cells significantly increases compared with that of normal eggs [28], indicating that giant eggs increase the ovoid shape by increasing the number of follicular cells during eggshell formation. Hence, the occurrence of giant eggs is a passive reaction caused by the increase in the egg contents.

The results of this study showed that the mutation of *PHYHD1* remarkably downregulated the *ESP* and some chorion genes, but ESP and chorion proteins did not decrease with the downregulation of gene expression. The mechanism of this process needs further study.

## Supporting information

S1 Table Primers used in screening candidate genes, sgRNAs synthesis and qRT-PCR. (PDF)

S2 Table DEGs between Yun7 and Yun7*Ge*. (PDF)

S3 Table DEGs of chorion protein genes with FPKM<100. (PDF)

S1 Fig The mutation sites at both ends of the mutation region. (PDF)

S2 Fig Agarose gel electrophoresis of chromosome walking. (A) Nested PCR was performed with specific primers SP1, SP2, SP3 and AP1, AP2, AP3, AP4 primers in Genome Walking Kit, respectively. The 3rd PCR product of AP4 was recovered for cloning and sequencing. Lanes 1–3 were the 1st, 2nd and PCR products of AP1, lanes 4–6 were the 1st, 2nd and 3rd PCR products of AP2, lanes 7–9 were the 1st, 2nd and 3rd PCR products of AP3, lanes 10–12 were the 1st, 2nd and 3rd PCR products of AP4, and lanes 13–15 are the 1st, 2nd and 3rd d PCR products of the positive control. (B) Nested PCR was performed with specific primers SP4, SP5, SP6 and AP1, AP2, AP3, AP4 primers in Genome Walking Kit, respectively. No PCR product was obtained in all combinations.
(PDF)

S3 Fig PCR amplification results using primers ScgF1 and ScgR with yun7GE genome as template. (PDF)

S4 Fig SNP analysis of the eggs laid by F1 generation. Primers were designed on both sides of the sgRNA site for PCR amplification, and the PCR products were subjected to SNP typing. The yellow curve represents the individuals with differences.
(PDF)

S1 Sequence The nucleotide sequence marked in red is the 3105bp sequence after mutation. (PDF)

## References

[1] Chen Z, Nohata J, Guo H, Li S, Liu J, Guo Y, et al. A comprehensive analysis of the chorion locus in silkmoth. Sci Rep. 2015; 5: 16424. doi: 10.1038/srep16424 26553298 PMC4639761

[2] Li B, Xia Q, Hirishi F, Yutaka B, Lu C. Fluorescent Differential Display of Kidney Egg Mutation (*ki*) from the Silkworm (*Bombyx mori*). Journal of Agricultural Biotechnology. 2002; 10 (3): 262–266.

[3] Liu X, Ma X, Yi X, Hou C, Li M. Fine Mapping of Bombyx mori Ellipsoid Egg Gene elp and Preliminary Analysis of Candidate Genes. Sci Seric. 2014; 40 (2): 0344–0347.

[4] Dai F, Wang X, Hu H, Lu C, Xiang Z. The Specific Character and Heredity of Mutant New Small Egg (*sm-n*) in the Silkworm. Sci Seric. 2006; 32 (4): 459–463.

[5] Chen A, Zhao Q, Zhang G, Qiu Z, Xia D, Dai F. Eggshell Structure and Genetic Analysis of Lethal Egg Mutation (*l-e*^*m*^) in Silkworm Variety “ming”. Sci Seric. 2009; 35(1):139–143.

[6] Chen A, Gao P, Zhao Q, Tang S, Shen X, Zhang G, et al. Mutation of a vitelline membrane protein, BmEP80, is responsible for the silkworm “Ming” lethal egg mutant. Gene. 2013; 515(2): 313–319. doi: 10.1016/j.gene.2012.12.006 23262333

[7] Xiang Z. Genetics and breeding of *Bombyx mori*. China Agriculture Press, 1994.

[8] Kawamoto M, Jouraku A, Toyoda A, Yokoi K, Minakuchi Y, Katsuma S, et al. High-quality genome assembly of the silkworm, *Bombyx mori*. Insect Biochem Mol Biol 2019; 107: 53–62. doi: 10.1016/j.ibmb.2019.02.002 30802494

[9] Kawaguchi Y, Shito K, Fujii H, Doira H. Manifestation of characters in the “giant egg” mutant of *Bombyx mori* (Lepidoptera: Bombycidae), 1: characteristics of the *Ge* egg. Jpn. J. Appl. Ent. Zool. 1987; 31(4): 344–349.

[10] Tang S, Shen X, Zhao Q, Zhang Y, Zhang G, Guo X. Studies on Pretreatment of Eggs From Bivoltine Silkworm Varieties (Strains) for Micro-injection Transgene. Sci Seric. 2009; 35(4):872–876.

[11] Trapnell C, Pachter L, Salzberg SL. TopHat: discovering splice junctions with RNA-Seq. Bioinformatics. 2009; 25(9): 1105–1111. doi: 10.1093/bioinformatics/btp120 19289445 PMC2672628

[12] Mortazavi A, Williams BA, McCue K, Schaeffer L, Wold B. Mapping and quantifying mammalian transcriptomes by RNA-Seq. Nature methods. 2008; 5(7): 621–628. doi: 10.1038/nmeth.1226 18516045 PMC13303166

[13] Wang L, Feng Z, Wang X, Wang X, Zhang X. DEGseq: an R package for identifying differentially expressed genes from RNA-seq data. Bioinformatics. 2010; 26(1): 136–138. doi: 10.1093/bioinformatics/btp612 19855105

[14] Shannon P, Markie A, Ozier O, Baliga NS, Wang JT, Ramage D, et al. Cytoscape: a software environment for integrated models of biomolecular interaction networks. Genome Res. 2003; 13(11): 2498–2504. doi: 10.1101/gr.1239303 14597658 PMC403769

[15] Götz S, García-Gómez JM, Terol J, Williams TD, Nagaraj SH, Nueda MJ, et al. High-throughput functional annotation and data mining with the Blast2GO suite. Nucleic Acids Res. 2008; 36(10): 3420–3435. doi: 10.1093/nar/gkn176 18445632 PMC2425479

[16] Zhao Q, Zhang Z, He J. A rapid method for preparing genomic DNA from pupae, *Bombyx mori*. Sci. Seric. 2000; 26(1): 63–64.

[17] Wang P, Zhao Q, Qiu Z, Bi S, Wang W, Wu M, et al. The silkworm (*Bombyx mori*) neuropeptide orcokinin is involved in the regulation of pigmentation. Insect Biochem Mol Biol. 2019; 114:103229. doi: 10.1016/j.ibmb.2019.103229 31449846

[18] Mounier N, Prudhomme JC. Isolation of actin genes in *Bombyx mori*: the coding sequence of a cytoplasmic actin gene expressed in the silk gland is interrupted by a single intron in an unusual position. Biochimie. 1986; 68(9): 1053–1061. doi: 10.1016/s0300-9084(86)80179-1 3096383

[19] Livaka KJ, Schmittgen TD. Analysis of Relative Gene Expression Data Using Real-Time Quantitative PCR and the 2^-ΔΔC^_T_ Method. Methods. 2001; 25(4): 402–408. doi: 10.1006/meth.2001.1262 11846609

[20] Fujii T, Abe H, Kawamoto M, Banno Y, Shimada T. Positional cloning of the sex-linked giant egg (*Ge*) locus in the silkworm, *Bombyx mori*. Insect Molecular Biology. 2015; 24(2): 213–221. doi: 10.1111/imb.12150 25469867

[21] Paul M, Goldsmith MR, Hunsley JR, Kafatos FC. Specific protein synthesis in cellular differentiation: Production of eggshell proteins by silkmoth follicular cells. J Cell Biol. 1972; 55(3): 653–680. doi: 10.1083/jcb.55.3.653 4656706 PMC2108812

[22] Swevers L, Iatrou K. The ecdysone regulatory cascade and ovarian development in lepidopteran insects: insights from the silkmoth paradigm. Insect Biochem Mol Biol. 2003; 33(12): 1285–1297. doi: 10.1016/j.ibmb.2003.06.012 14599500

[23] Blau HM, Kafatos FC. Secretory kinetics in the follicular cells of silkmoths during eggshell formation. J Cell Biol. 1978; 78(1): 131–151. doi: 10.1083/jcb.78.1.131 566758 PMC2110171

[24] Sato Y, Yamashita O. Structure and expression of a gene coding for egg-specific protein in the silkworm, *Bombyx mori*. Insect Biochem. 1991; 21(5): 495–505.

[25] Izumi S, Yano K, Yamamoto Y, Takahashi SY. Yolk proteins from insect eggs: structure, biosynthesis and programmed degradation during embryogenesis. J. Insect Physiol. 1994; 40(9): 735–746.

[26] Zhang Z, Kochan GT, Ng SS, Kavanagh KL, Oppermann U, Schofield CJ, et al. Crystal structure of PHYHD1A, a 2OG oxygenase related to phytanoyl-CoA hydroxylase. Biochem Biophys Res Commun. 2011, 408(4): 553–558. doi: 10.1016/j.bbrc.2011.04.059 21530488

[27] Yokoi K, Tsubota T, Sun J, Jouraku A, Sezutsu H, Bono H. Reference transcriptome data in silkworm *Bombyx mori*. 2019; BioRxiv preprint 10.1101/805978. PMC8228281 34205145

[28] Kawaguchi Y, Banno Y, Koga K, Doira H, Fujii H. Appearance of “Giant Egg” Mutant of *Bombyx mori* (Lepidoptera: Bombycidae). 4. Surface Structure of Eggshell in *Ge* egg. Jpn. J. Appl. Ent. Zool. 1993; 37(2): 91–95.

