# Supplementary material for: phytanoyl-CoA dioxygenase domain-containing protein 1 plays an important role in egg shell formation of silkworm (Bombyx mori)
Source: PLoS One. 2021 Dec 30;16(12):e0261918. doi: 10.1371/journal.pone.0261918 (PMC8717975; doi:10.1371/journal.pone.0261918)
Supplement: S1 Table — (PDF) [file pone.0261918.s001.pdf]

Table S1 Primers used in screening candidate genes, sgRNAs synthesis and qRT-PCR

Purpose of primers	Primer name	Primer sequence
Screening candidate genes	ScgF	AACACGAACGGTACTTCGCT
	ScgR	CGCAAGGTCAAGGAATTGTG
	ScgF1	GGAAGACTAGCTTGCATGGAT
Chromosome walking	SP1	CCTAACACGAACGGTACTTCGCTG
	SP2	ATCAGAATCGCGATCCACTGAGAAG
	SP3	GAGCGCCTCTGAAGGAGACCTAAC
sgRNAs synthesis	*PHYHD1*-sgRNA1	TTCTAATACGACTCACTATAGtgaaatcaaagccgctggatGTTTTAGAGCTAGA
	PHYHD1-sgRNA2	TTCTAATACGACTCACTATAgttgctctctcctctatgtcGTTTTAGAGCTAGA
qRT-PCR	KWMTBOMO00542	Forward: AGCTCTTGAAGAAGCCACAG<br>Reverse: GGAGCTCTTATGTACAACGT
	KWMTBOMO00765	Forward: GTCTTAGTAGACCTTGCAAC<br>Reverse: ACACCAATGGACTTGAAGGT
	KWMTBOMO01504	Forward: CCTTGCTGTGGTGACTTCTT<br>Reverse: GAGCATAGTTAATGGCAGGA
	BMgn014786	Forward: TGTGTACAGTCAGTGCTTGG<br>Reverse: AGATGGAGACAGAACCGACA
	KWMTBOMO00759	Forward: TACACAGTCAGTGCTTAGGT<br>Reverse: GTTATTGGTGATGTACTGGT
	KWMTBOMO00758	Forward: CCAGTCTGCTGTCAGTCAAT<br>Reverse: ACACCTAAACTAGTAGGAGC
	KWMTBOMO00740	Forward: TCCAAACGGCTTAGCAGCTT<br>Reverse: CTCCACATCCATAAGTTACAG
	BMgn014782	Forward: AACGGATACAGTGGTCCCAT<br>Reverse: TTCCGCTGATCGTCACAGAT
	KWMTBOMO00757	Forward: CGCTAACTTAGCAGCATCAT<br>Reverse: GCTTCATTCGTGATGCCAAT
	KWMTBOMO00763	Forward: TCGGTGATGATATCGCAGCT<br>Reverse: ATACCACCAACACCAGCAGT
	BMSK0000832	Forward: CTGCTCTTGAAGCTTCTCGT<br>Reverse: TACCGAGGAGAGGCAGGTT

Note: The underlined sequence is the T7 promoter, and the lowercase letters are the sgRNA sequence.
