# Supplementary material for: phytanoyl-CoA dioxygenase domain-containing protein 1 plays an important role in egg shell formation of silkworm (Bombyx mori)
Source: PLoS One. 2021 Dec 30;16(12):e0261918. doi: 10.1371/journal.pone.0261918 (PMC8717975; doi:10.1371/journal.pone.0261918)
Supplement: S2 Table — (PDF) [file pone.0261918.s002.pdf]

Table S2 DEGs between Yun7 and Yun7$^{Ge}$

| Gene name in NCBI | Gene name | Chromosome | log2(Yun7$^{Ge}$_1/Yun7_1) | log2(Yun7Ge_2/Yun7_2 | Up/Down | Description |
|---|---|---|---|---|---|---|
| LOC101735439 | KWMTBOMO00542 | 1 | -12.6281 | -14.3189 | down | phytanoyl-CoA dioxygenase domain-containing protein 1 |
| LOC101742372 | BMSK0000826 | 2 | -1.89536 | -2.24775 | down | chorion class CB protein M5H4-like |
| LOC101739564 | BMgn014782 | 2 | -2.27046 | -2.48944 | down | chorion class CA protein ERA.1-like |
| LOC101739706 | KWMTBOMO00756 | 2 | -2.01117 | -2.31442 | down | chorion class B protein PC10 |
| LOC101739848 | KWMTBOMO00757 | 2 | -1.66414 | -2.08074 | down | chorion class CB protein M5H4-like |
| LOC101740116 | KWMTBOMO00758 | 2 | -2.61037 | -3.59843 | down | chorion class B protein M3A5-like |
| LOC101740255 | KWMTBOMO00759 | 2 | -1.31384 | -2.03465 | down | chorion class CB protein M5H4-like |
| LOC101740388 | BMgn014783 | 2 | -1.2589 | -2.32325 | down | chorion class B protein ERB4-like |
| Era.3 | BMgn014786 | 2 | -1.77008 | -2.31155 | down | chorion protein gene ErA.3 |
| Era.2 | BMgn014787 | 2 | -1.69236 | -2.10898 | down | chorion protein gene ErA.2 |
| Erb.2 | KWMTBOMO00763 | 2 | -1.27043 | -2.10866 | down | chorion protein gene ErB.2 |
| Erb.1 | KWMTBOMO01504 | 2 | -2.51164 | -2.53556 | down | chorion protein gene ErB.1 |
| Era.1 | KWMTBOMO00762 | 2 | -1.5522 | -2.11409 | down | chorion protein gene ErA.1 |
| 5h4 | KWMTBOMO00764 | 2 | -1.46789 | -1.82612 | down | chorion CB early protein gene 5H4 |
| LOC101740521 | KWMTBOMO00765 | 2 | -1.15205 | -2.1511 | down | chorion class B protein ERB4-like |
| LOC101740806 | KWMTBOMO00766 | 2 | -1.30992 | -2.36089 | down | chorion class B protein ERB4-like |
| LOC101741090 | KWMTBOMO00772 | 2 | -2.60983 | -3.13529 | down | chorion class B protein ERB4-like |
| LOC101742521 | KWMTBOMO00770 | 2 | -1.78188 | -2.21771 | down | chorion class CB protein M5H4-like |
| LOC101741649 | Undetected | 2 | -2.10405 | -1.25398 | down | chorion class A protein L12 |
| LOC101742957 | BMSK0000832 | 2 | -1.57639 | -1.18652 | down | chorion class B protein L11-like |
| LOC101740390 | KWMTBOMO00753 | 2 | -2.97185 | -4.25184 | down | chorion class A proteins Ld9 |
| LOC101740951 | BMSK0000822 | 2 | -1.27535 | -1.57195 | down | chorion class CA protein ERA.3-like |

| | | | | | | |
|---|---|---|---|---|---|---|
| LOC101740807 | BMSK0000824 | 2 | -1.14854 | -1.78546 | down | chorion class CA protein ERA.2-like |
| LOC101744169 | Undetected | 2 | -1.24042 | -2.52514 | down | chorion class A protein L12 |
| LOC101736313 | KWMTBOMO00740 | 2 | -4.40773 | -4.66333 | down | chorion class CB protein M5H4-like |
| LOC101736796 | KWMTBOMO00738 | 2 | -4.8536 | -5.84193 | down | uncharacterized |
| LOC101737055 | KWMTBOMO00737 | 2 | -2.05538 | -2.71399 | down | TBC1 domain family member 5 homolog A |
| LOC101737341 | BMSK0000797 | 2 | -6.4533 | -7.59804 | down | uncharacterized |
| LOC101737482 | KWMTBOMO00736 | 2 | -2.47915 | -3.43055 | down | keratin-associated protein 10-7 |
| LOC101737906 | KWMTBOMO00734 | 2 | -6.24593 | -7.34716 | down | uncharacterized |
| LOC101738165 | KWMTBOMO00733 | 2 | -5.36941 | -5.90973 | down | uncharacterized |
| LOC101738300 | BMgn014773 | 2 | -5.03603 | -7.05275 | down | uncharacterized |
| LOC101738437 | KWMTBOMO00732 | 2 | -5.54587 | -5.63262 | down | MATH and LRR domain-containing protein PFE0570w |
| LOC101738566 | BMSK0000792 | 2 | -2.93138 | -4.57841 | down | uncharacterized |
| LOC101738700 | BMSK0000791 | 2 | -2.83146 | -3.96557 | down | uncharacterized |
| Jhdk | KWMTBOMO01581 | 3 | -3.15224 | -4.17263 | down | juvenile hormone diol kinase |
| LOC101742363 | KWMTBOMO02196 | 4 | 4.65367 | 2.02322 | up | clavesin-1 |
| LOC101742655 | KWMTBOMO02193 | 4 | -1.42303 | -1.2776 | down | alpha-tocopherol transfer protein-like |
| LOC732862 | KWMTBOMO03113 | 5 | -1.20309 | -1.00518 | down | signal sequence receptor beta subunit |
| LOC101737921 | KWMTBOMO05573 | 10 | -5.90857 | -5.28002 | down | VMP25 |
| LOC101737788 | KWMTBOMO05574 | 10 | -5.65456 | -1.32335 | down | uncharacterized |
| LOC101738054 | KWMTBOMO05575 | 10 | -8.59884 | -1.33292 | down | asparagine-rich protein |
| LOC101736547 | KWMTBOMO06665 | 11 | -6.90908 | -2.68877 | down | gamma-glutamyl hydrolase A |
| LOC101739808 | KWMTBOMO07532 | 12 | 3.06486 | 1.35408 | up | ATP-binding cassette sub-family G member 1 |
| LOC101742779 | KWMTBOMO07500 | 12 | -8.39358 | -9.5078 | down | uncharacterized |
| LOC101744925 | KWMTBOMO09222 | 13 | -6.83599 | -4.98497 | down | uncharacterized |

| | | | | | | |
|---|---|---|---|---|---|---|
| LOC733141 | KWMTBOMO07600 | 13 | -1.19489 | -1.05038 | down | transport protein Sec61 gamma subunit |
| LOC101738705 | KWMTBOMO07599 | 13 | -1.09105 | -1.49481 | down | glycine N-methyltransferase |
| LOC101738784 | KWMTBOMO09157 | 15 | -1.93019 | -2.46965 | down | leucine-rich repeat extensin-like protein 5 |
| LOC105842955 | KWMTBOMO09157 | 15 | -3.86905 | -4.85793 | down | uncharacterized LOC105842955 |
| LOC101741055 | KWMTBOMO09108 | 15 | -7.39453 | -1.45199 | down | follicle cell protein 3C-1 |
| Mmp1 | KWMTBOMO08941 | 15 | 2.06494 | 1.33676 | up | matrix metalloproteinase 1 |
| LOC101746850 | KWMTBOMO09843 | 16 | -3.25015 | -4.43132 | down | leucine-rich repeat extensin-like protein 3 |
| LOC101747122 | BMSK0009512 | 16 | -5.11869 | -5.49539 | down | extensin |
| LOC101743035 | KWMTBOMO09561 | 16 | 1.29192 | 1.40187 | up | aldo-keto reductase AKR2E4 |
| LOC692581 | KWMTBOMO10249 | 17 | -7.95387 | -5.90311 | down | vitelline membrane associated protein P30 |
| LOC693022 | KWMTBOMO11748 | 19 | -9.72199 | -2.46143 | down | egg-specific protein |
| LOC101739782 | KWMTBOMO12298 | 20 | 3.01991 | 1.58353 | up | uncharacterized |
| LOC101745618 | KWMTBOMO12167 | 20 | 1.0887 | 1.11213 | up | RNA-binding protein pno1 |
| Bcp | KWMTBOMO13997 | 23 | 2.25271 | 1.56248 | up | fibroinase |
| LOC101740215 | KWMTBOMO15050 | 25 | -1.2766 | -1.37667 | down | adenylosuccinate synthetase |
| LOC101744931 | KWMTBOMO15166 | 25 | -1.53882 | -2.35318 | down | cystathionine beta-synthase |
| LOC733007 | KWMTBOMO14914 | 25 | -1.22706 | -1.27449 | down | copper transporter |
| LOC101744536 | KWMTBOMO16199 | 28 | -3.33183 | -1.34554 | down | uncharacterized |
| GSTd2 | KWMTBOMO16484 | Unplaced | -1.45181 | -1.58007 | down | glutathione S-transferase delta 2 |

Note: The genes named KWMTBOMO, BMSK and BMgn were derived from http://sgid.popgenetics.net/, https://silkdb.bioinfotoolkits.net/ and https://kaikobase.dna.affrc.go.jp/. Undetected means genes that had not been detected in the above websites.
