# Supplementary material for: phytanoyl-CoA dioxygenase domain-containing protein 1 plays an important role in egg shell formation of silkworm (Bombyx mori)
Source: PLoS One. 2021 Dec 30;16(12):e0261918. doi: 10.1371/journal.pone.0261918 (PMC8717975; doi:10.1371/journal.pone.0261918)
Supplement: S3 Table — (PDF) [file pone.0261918.s003.pdf]

Table S3 DEGs of chorion protein genes with FPKM<100

Gene name in NCBI	Gene name	Chromosome	log2(Yun7^{Ge}_1/Yun7_1)	log2(Yun7Ge_2/Yun7_2	Up/Down	Description
C/EBP	KWMTBOMO11827	2	-1.66432	-1.09099	down	chorion specific C/EBP
Cbz	KWMTBOMO03312	2	0.374682	0.512084	up	chorion b-ZIP transcription factor
Era.4	KWMTBOMO00760	2	-0.33382	-0.71692	down	chorion protein gene ErA.4
Era.5	KWMTBOMO00768	2	-0.998153	-2.02714	down	chorion protein gene ErA.5
Erb.3	KWMTBOMO00761	2	-0.83352	-1.88943	down	chorion protein gene ErB.3
LOC101735781	Undetected	2	-0.65133	-0.92194	down	chorion class A protein L12-like
LOC101736040	Undetected	2	-0.899549	-0.64004	down	chorion class A protein L12-like
LOC101736564	KWMTBOMO00739	2	-0.644736	-0.42144	down	chorion class CA protein ERA.2-like
LOC101738105	Undetected	2	-0.503948	-1.17854	down	chorion class B protein M2410-like
LOC101739985	KWMTBOMO00758	2	-0.889238	-1.55766	down	chorion class B protein M3A5-like
LOC101740257	BMgn014779	2	-1.09243	-2.47152	down	chorion class B protein Ld34-like
LOC101740523	KWMTBOMO00754	2	-0.135797	-0.37263	down	chorion class B protein M2410-like
LOC101740949	KWMTBOMO00767	2	-0.973458	-1.43173	down	chorion class CB protein M5H4-like
LOC101741014	Undetected	2	0.516416	0.619171	up	chorion class A protein L11-like
LOC101741368	KWMTBOMO03269	2	-2.91834	-3.10614	down	chorion peroxidase-like
LOC101742258	Undetected	2	-0.974574	-1.62354	down	chorion class B protein M3A5-like
LOC101742663	Undetected	2	-1.98212	-0.94609	down	chorion class A protein L12-like
LOC101742812	KWMTBOMO00771	2	-1.87558	-0.15611	down	chorion class B protein M2410-like
LOC101742834	Undetected	2	-0.51953	-0.46926	down	chorion class B protein M3A5-like
LOC101742977	KWMTBOMO00744	2	0.720617	0.504854	up	chorion class B protein M2807-like
LOC101743021	Undetected	2	-0.481024	-1.27064	down	chorion class B protein M3A5-like
LOC101743094	Undetected	2	-1.33643	-0.46106	down	chorion class A protein L12-like
LOC101743307	Undetected	2	-1.00113	-0.00787	down	chorion class B protein M2410-like

LOC101743449	Undetected	2	-0.284669	-0.45818	down	chorion class A protein L12-like
LOC105841460	Undetected	2	-0.990224	-2.04263	down	chorion class B protein M2410-like

Note: The genes named KWMTBOMO, BMSK and BMgn were derived from http://sgid.popgenetics.net/, https://silkdb.bioinfotoolkits.net/ and https://kaikobase.dna.affrc.go.jp/. Undetected means genes that had not been detected in the above websites.
