# Supplementary figures and images for: phytanoyl-CoA dioxygenase domain-containing protein 1 plays an important role in egg shell formation of silkworm (Bombyx mori)
Source: PLoS One. 2021 Dec 30;16(12):e0261918. doi: 10.1371/journal.pone.0261918 (PMC8717975; doi:10.1371/journal.pone.0261918)

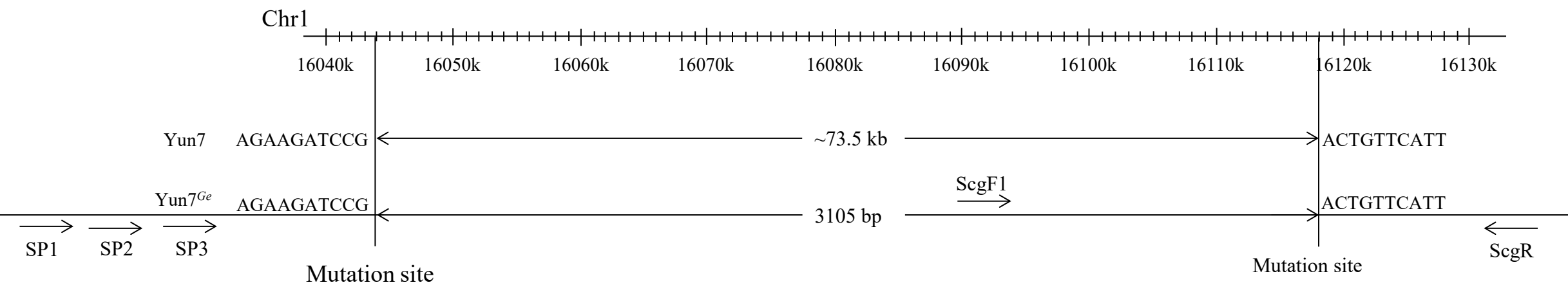

S1 Fig

Supplement: S1 Fig — (PDF) [file pone.0261918.s004.pdf]

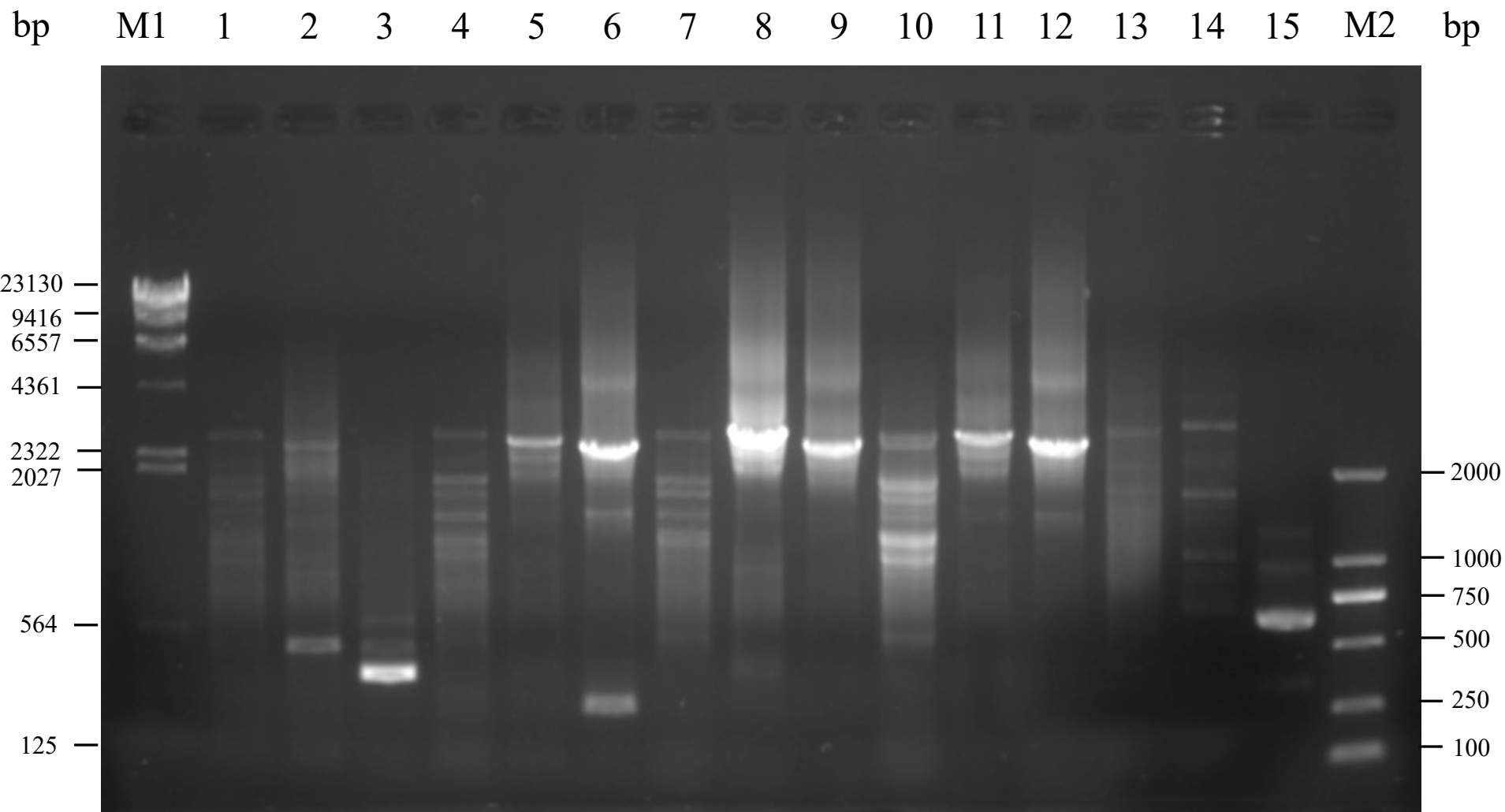

S2 Fig

Supplement: S2 Fig — (A) Nested PCR was performed with specific primers SP1, SP2, SP3 and AP1, AP2, AP3, AP4 primers in Genome Walking Kit, respectively. The 3rd PCR product of AP4 was recovered for cloning and sequencing. Lanes 1–3 were the 1st, 2nd and PCR products of AP1, lanes 4–6 were the 1st, 2nd and 3rd PCR products of AP2, lanes 7–9 were the 1st, 2nd and 3rd PCR products of AP3, lanes 10–12 were the 1st, 2nd and 3rd PCR products of AP4, and lanes 13–15 are the 1st, 2nd and 3rd d PCR products of the positive control. (B) Nested PCR was performed with specific primers SP4, SP5, SP6 and AP1, AP2, AP3, AP4 primers in Genome Walking Kit, respectively. No PCR product was obtained in all combinations. (PDF) [file pone.0261918.s005.pdf]

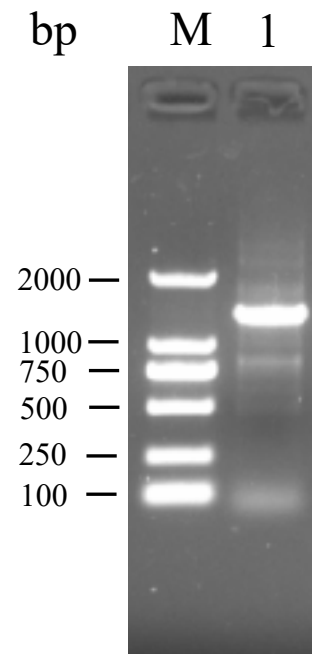

S3 Fig

Supplement: S3 Fig — (PDF) [file pone.0261918.s006.pdf]

Temperature-Shifted Difference Curve

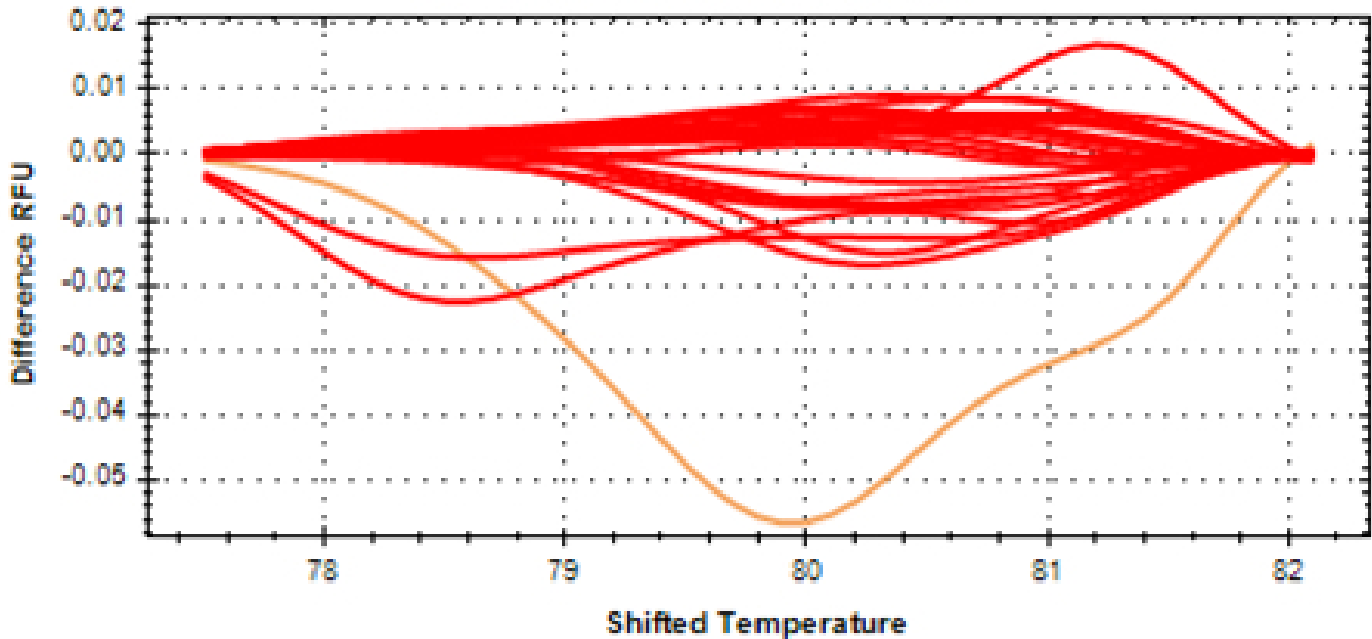

S4 Fig

Supplement: S4 Fig — Primers were designed on both sides of the sgRNA site for PCR amplification, and the PCR products were subjected to SNP typing. The yellow curve represents the individuals with differences. (PDF) [file pone.0261918.s007.pdf]
