# Supplementary material for: phytanoyl-CoA dioxygenase domain-containing protein 1 plays an important role in egg shell formation of silkworm (Bombyx mori)
Source: PLoS One. 2021 Dec 30;16(12):e0261918. doi: 10.1371/journal.pone.0261918 (PMC8717975; doi:10.1371/journal.pone.0261918)
Supplement: S1 Sequence — (PDF) [file pone.0261918.s008.pdf]

The nucleotide sequence marked in red is the 3105bp sequence after mutation. The underlined sequence is the position of the primers, and the name of the primers is in parentheses.

TGGTAGCTGGTAGCCTAGAAGGTTATTTAATCTACGCCCTGACGGGTAATTGAGCTCAC
GTGCTCGAAAGAATTTACTAACATCAGCCCTAACACGAACGGTACTTCGCTG(ScgF,SP1)
AATCTACCACCGGATCAGAATCGCGATCCACTGAGAAG(SP2)ACCCAACAAAAAACTC
AGTCGGTTCCTGAATGATTTGTACGTTTACAGTTTACGGCTTATACTGCCTATTTGTTA
TTTATTACAGTCTTTTCTTTTCTTTTCTTTTTTTTTATGATTGATAGTTTACTGGTGGCCCG
AAGGCCTTTCCAGTTTCACCAGGACAGGTGGGCGAGCAAAGGCTCAGCCAAGAGGGG
CGGGATTTGCTAACAACTGCCCGAGCGCCTCTGAAGGAGACCTAAC(SP3)AACTCAAG
AGCAATTGCTTCGCGAATGAATCTACTACCGGATCGGAATCGCGACCCGCTGAGAAGA
TCCGACTAGGCTGCCCGGACCAGGTCACGGATACGACCCCGGTCCCGACCCCCTGCTC
GGCGGCGGCGGGTTTCTGCGTAGTGAGGAGAGCTTTCCCTCACTCGCCCCGCCGCCTC
CTTCTGCGAGATGGTGCACTCGCAGAAGTCCAGCATAGCCTTCCATGACTCATCGCTGC
CAAGCATCGACGCCACGACGCCAGGCAGCGACAAGTCAGGTCCTATCTTTGCGACCAC
GACACGGCGCTGCACCTCCCAAGCGGGGCAGACAGCGAGCGTATGCTCCGCCGTGTC
CAGGTCGTGTCCACAATGGTGACACCTCGTCGTCGGCTCAGCCCCTATCCGGCGCAGG
TACTTCCCGAAGCATCCGTGCCCGGTCAGCACCTGCACCAGACGAAAGGTGAGACGT
CCTTCGCCACGATTCACCCAGTCATCAAAGACCGGGTAAACCGCCTCGACGGTCCGTA
GCCCCCACGTGGGGTTGGCCAATCGCCTCGACCACGACTCGATTCTATTTCTATTTAGT
TTGTTATCAACAAATGTCGCTGCACGTAAATTCAACAATTCCTGAATCGCGGTGACGAG
ATGTTACACAGTTGATAGACGTTCTCGTATTCCTCTTGCTAAATCATACCTACCTGGGAC
AGACATCGGGATACCGTGCCTGGCATCGGGATCATGCAGGCGGTTCCTCCAGGGTGCG
GCAGCTCCATTGCACTGCGGAGTGGTTGACCCTTGCGATTATTTTATACTTCTACCGTG
TGTGACATGCACAGACACATTTTTTTTAAATTATATTACAACATTTTTATGTAATGCTTTT
TGGCAAGCTATAAGAAAGGCAAGACAAAACAAATTTAACAGAATGTAAACATAAGCCT
ATATTTCGTGAATTGCAATCTAAACCAAACTTAGTAGAATCTCCAACATCACTACTACTA
CTCCGGTGGCGCTAGGACCCCGAAGTGGGTCTTGGCCTCCGACAATGAACGCCGCCAT
CCATCCCGGCGCTGTGCAGCACTATGCCAGTTCGGCACTTGGAGCCCGCGTAGATCCT
CTTCCACAACGTCAGACCAGCAGTACTTAGGTCTCCCTACGGGGCGACGTCCCTCTGG
TACCCCCACTTCACAGCCCGAACCCCTACACTAAATGTCCGAGCCACGTGGACCTCCG
CGCCTTCCTCTCTCGTAGAATATTTGGCTTACCAAAAAGATTCTGAACATATTTTTTGTT
CTTCTGGAAGAACAAAAAAGATGCCAACGTGCCAACCAACATTATCTGGGACTGGCCC
CAGTATCTTTCTCAGTACCTTTCGCTCAATTCGCTCGCGTAGTCGTCGTGGCCTAAAGG
ATAAGACCTCCGGTGATAAGACCGAACACCGGCGCATTCGCATTGAGCGATGCACCGG
TGTTCGAATCTCAGGCGGGTACCAATTTTTCTGATGAAATACGTACTCAACAAATGTTC
ATGATTGACTTCCACAGTAAAGGAATAACATCGTGTATAAAAATTAATCCTGCAAAATTA
TAATTTGCGTAATTACTTGTGGTAGGACCTATTGTTAGTCCGCGCGGCTAGATACCACCA
CCCTGCCTATTTCTGACGTGAAGCAGTAATGCGTTTCGGTTTGAAGGGGTAGCCGTTGT
AACTATACTGAGACCTTACAACTTATGTCTCAAGGTGGATGGCGCATTTACGTTATAGAT
GTCTTTGGGTTCCAGTAACCACTTAACACCAGGTGGGCCGTTAGCTCGTCCACCCATGT
AAGCAATAAAAAAAAGTTACGATATGGTTTGCCTCTTCTTTCGATGTAAACCTTAATGC
TTCTCAACCATATAATAGTTTGACTACGGTCTTGTATATCCGAATGCTGGTGCGTCTGCTT
AGTAACTTTGAAGAGATCTGCATGCTGGGCCTTCGGGTAGGCTGACGGGTGGCGCTTC

TCTGTCATTCTCTGTTACAGTGCAGCCAAGGTACCGGATTTTGGTCTGAAGTTTTCTCG
CTTCCGTCTTGTGTTTTTTTATGGACGCGTGTGAAGATACATCGTCTTTTCGTGGCTCAC
ACGAAGGCCAATCAGAGAGGCTTTCTGTTTTAGCCAATTTAATGCATCTCCTAGTAAGG
CCATCGGCATACTCTATCATCTTATGTTGTCCATTGAGCTGCAATCCATTAATACAAGTGT
GTACATATTCCGTGTAACGTGTACAAGTACCAGGTTGGACCATATCACAAGAAAATATT
TGGTAATATTAAACAGGTTTTGTTTATTTGTGTAGATTATGAGGAAGACTAGCTTGCATG
GAT(ScgF1)CTTATTAAGCGGCTTCGAATAATAATTTGAAATATGAGATCGAAGTTAAATA
CATAAAATTACAATTTATAATGAATATCTGAAATGAAATTTCAAATACATACTTAACAAA
GAAGCTGTTCTTCTAAATTACAATGTTGATAAGCAATGTTTCTTACAAACCATGTTTTCT
ATTCATTAAAATCTAAGAAGAAAACTGTTTTGTAAGTTTTTTTTTAATACATTGCATGA
AAAGGGCTTATTACTTCGTATAAAATTTTTAAAAGACCAAAGCAGACTAATTCGTTATTA
AAAGCTTAAAAAAAACATAATACGAATAACATAAACTTAGCCAAATAGAATACGAACG
CTGAACTTTGCAAAGTGTTGAGTTCATTTAGAAAAGAGATAAGTAGTTGTAATTATTTT
AAATAATGCTATAGATACATAAATAATATTGAGAGAGAGATAAATTTTAATATTAGAGGT
GGGGCATTTTAAAACCCTTGTTAGATTCGATATAACGAGAGGAATAGTATCGATTCGTAA
AATAACTAAACAACCAAATTATTTAAAAAATACACATGCGGTATCTGTACAGGGCACAT
TTATTTTAATCATTCTTTCAATAATTATTGTAATAGCTATCATCACGCAAAATATATGTGGG
GTCGCGGACTTCGGGACACGAACCTAATGTCGATATTTAAAGATTCAACATATATTTATA
TATGAATGTATCCACTGCAACGCTCACTATAACATAATCTACTTTTTGTCAATGAATGCT
GCTATTTTTTTCAATTAAATTCGAGCTTCGCATCAGGAACTGTTCATTTTAGATTTATTT
TTATTTTGTTCGTTTTGTTTTCGATTTCCTATCAAGACACTACGGTCGGGCAGGGTCAG
TTACAATTCGCTATTCATGAAATGAGATTATCCTGATTTATTTAAGGTTGATCATATTT
GAAAGCGTATTTTAACTGCTTACAGTATTAAATAATATTTGAGATGGCGACAAACAAC
ACCAACGGGCATTGCGAGAACTACCGTGAGTTTTTAATTCTACCGTACACGTATTTGT
TTGCGAACCAAAAGTTTTTCGCTTTTCGCTTTGATTATTGATTGATGCAGATGCGTGCA
GTTGCAGCCATTGTGAAATGTGATGAGTACCTTTTGAATCTTAAACGCTTCTCTTGTTC
TACTTAAAATTAACATTCCCCGTTATTACCAGTTTACTGACGCAGTAATTTAATGATA
CTAAATGATTTTGATCATTTAAAAAAAATCAATTCATCAATAAATTACATTTTAATAA
TGAAAAATTTCTGCAGCTATGTTGGTTAAAGCTCAATTAAATAGTGCGTAATTTTTTTT
TTGTTTCACATTATTTTTGAGCCTTTTTTATTTTAATGCCCTTGAACTTGTGCCACAATT
CCTTGACCTTGCG(ScgR)TTTCATTAAAAATCAATTCGGTGTGATTTTGCCTAAACCTA
AACTAATCGCATGACG
